# Female Genital Schistosomiasis as a Cause of Tubal Ectopic Pregnancy and Recurrent Pregnancy Loss: A Case Report

**DOI:** 10.1155/crip/7652671

**Published:** 2025-03-10

**Authors:** Dirar Medhanie Gebremedhin, Hale Teka, Kidan Fssaha Tsehaye

**Affiliations:** ^1^Department of Pathology, School of Medicine, Ayder Comprehensive Specialized Hospital, Mekelle University, Mekelle, Ethiopia; ^2^Department of Gynecology and Obstetrics, School of Medicine, Ayder Comprehensive Specialized Hospital, Mekelle University, Mekelle, Ethiopia; ^3^Department of Internal Medicine, School of Medicine, Ayder Comprehensive Specialized Hospital, Mekelle University, Mekelle, Ethiopia

**Keywords:** female genital schistosomiasis, ovarian schistosomiasis, recurrent pregnancy loss, *Schistosoma haematobium*, tubal ectopic pregnancy

## Abstract

**Background:** Schistosomiasis is a widespread parasitic disease that affects various organs, including the female genital tract. Female genital schistosomiasis can lead to significant reproductive morbidity, such as ectopic pregnancies and infertility.

**Case Presentation:** A 27-year-old woman with a history of recurrent spontaneous abortions presented with acute abdominal pain. She was diagnosed with a ruptured left ectopic pregnancy. Histopathologic examination of the resected tissue revealed numerous *Schistosoma haematobium* eggs within the ovarian parenchyma and fallopian tube, surrounded by granulomatous inflammation. The patient was treated with praziquantel and informed about the possible effects of schistosomiasis on her reproductive health.

**Conclusion:** This case emphasizes the importance of considering female genital schistosomiasis in women from endemic areas with ectopic pregnancies and recurrent pregnancy loss. Early diagnosis and treatment are essential to prevent long-term reproductive sequelae.

## 1. Introduction

Schistosomiasis, also known as bilharzia, is a parasitic disease caused by trematodes of the genus *Schistosoma*. It is the second most important parasitic disease after malaria in terms of socioeconomic and health significance [1]. Over 240 million people are infected worldwide, with more than 700 million people living in endemic areas [2]. The majority of cases occur in sub-Saharan Africa, where *Schistosoma haematobium* and *Schistosoma mansoni are* widespread [3].

*S. haematobium* is mainly associated with urogenital schistosomiasis and affects both the urinary and genital organs [4]. Female genital schistosomiasis (FGS) is an underrecognized manifestation of urogenital schistosomiasis, affecting an estimated 56 million women in endemic regions [5]. FGS can affect the cervix, vagina, vulva, uterus, fallopian tubes, and ovaries, leading to a spectrum of gynecological and reproductive health problems [6].

The pathogenesis of FGS involves the deposition of *S. haematobium* eggs in the genital tissue, which causes a chronic granulomatous inflammatory reaction [7]. This inflammation can lead to ulceration, mucosal bleeding, and fibrosis, affecting the structural integrity and function of the reproductive tract [8]. Clinical manifestations of FGS include vaginal discharge, bleeding, dyspareunia, pelvic pain, and lesions that may mimic sexually transmitted infections or malignancies [9].

The diagnosis of genital schistosomiasis presents challenges. Microscopic examination of stool and urine remains the gold standard diagnostic test, but it necessitates the presence of adult worms to produce eggs; serological tests can identify less advanced infections [10]. Currently, the standard diagnostic approach for urogenital schistosomiasis involves syringe filtration of urine and counting the eggs retained on the filter [11]. A diagnosis of urogenital schistosomiasis through histopathology is confirmed by identifying the characteristic eggs of the parasite, specifically *S. haematobium*, within tissue samples obtained from a biopsy of the affected urogenital tract, which will show the eggs surrounded by a granulomatous inflammatory reaction; this is regarded as the definitive diagnostic method when other techniques, such as urine microscopy, prove inconclusive [10].

FGS has a significant impact on women's reproductive health, including infertility, spontaneous abortions, and ectopic pregnancies [10, 11]. Inflammation and scarring of the fallopian tubes can impair tubal patency and function, increasing the risk of tubal infertility and ectopic implantation [12]. In addition, FGS is associated with an increased susceptibility to human immunodeficiency virus (HIV) infection due to mucosal dysfunction and immunological changes [13, 14].

Despite its high prevalence and impact, FGS remains underdiagnosed and underreported [15]. Challenges in diagnosis arise from nonspecific symptoms, limited awareness among healthcare providers, and lack of sensitive diagnostic tools [16]. Conventional methods, such as urine microscopy, may not detect genital involvement, so direct visualization or histopathological examination is often necessary [17].

This case report demonstrates a rare presentation of FGS that manifested as a ruptured ectopic tubal pregnancy in a woman with recurrent pregnancy loss. The incidental detection of *S. haematobium* eggs in ovarian and tubal tissue emphasizes the importance of considering FGS in the differential diagnosis of reproductive health problems in endemic areas. By raising awareness of this disease, we aim to improve detection, rapid diagnosis, and appropriate treatment to mitigate the impact on women's health.

## 2. Case Presentation

A 27-year-old nulliparous woman with a history of four prior miscarriages between the 8th and 12th weeks of pregnancy presented to Ayder Comprehensive Specialized Hospital, in Tigray, Ethiopia, with acute onset of severe abdominal pain that persisted for 2 days and intensified in the last 30 min before admission. She reported 8 weeks of amenorrhea coinciding with her last menstrual period and had experienced two episodes of vomiting and scanty vaginal spotting.

Her medical history was unremarkable, with no known chronic illnesses or previous operations. She denied any history of vaginal discharge, sexually transmitted infections, contraceptive use, or substance abuse. The patient lived in Tigray's regional capital and worked in the private sector.

On physical examination, she appeared acutely sick due to pain. The vital signs showed hypotension (blood pressure: 90/60 mmHg), normal heart beat (heart rate: 84 beats per minute), and a normal temperature (36.4°C). She had mild pallor of the conjunctivae. Abdominal examination showed distension with direct and rebound tenderness in the lower abdomen, and shifting dullness was positive, suggesting free intra-abdominal fluid.

Laboratory tests revealed a positive urine test for human chorionic gonadotropin (hCG) and moderate anemia (hemoglobin level of 9.0 g/dL). Serum level of beta hCG was not determined. Pelvic ultrasound showed an empty uterine cavity with an endometrial slit and a 4 × 3.5 cm echogenic mass in the left adnexal region. A gestational sac with a fetal pole with positive cardiac activity was visualized, corresponding to a crown-rump length of 8 weeks and 3 days. Free fluid was noted in the posterior cul-de-sac and paracolic gutters.

The clinical diagnosis of a ruptured left ectopic pregnancy was made. The patient underwent an exploratory emergency laparotomy. The intraoperative findings included approximately 600 mL of hemoperitoneum and a 4 × 4 cm ruptured left adnexal mass adherent to the posterior–lateral aspect of the uterus and involving the left fallopian tube and ovary. The fallopian tube was adhered to the mass, and the mass was excessively bleeding. Subsequently, the mass was resected and the specimen was sent for histopathologic examination. The right adnexa appeared normal. It should be noted that there is no laparoscopic service at the Department of Obstetrics and Gynecology at Mekelle University, College of Health Sciences.

**Figure 1:**
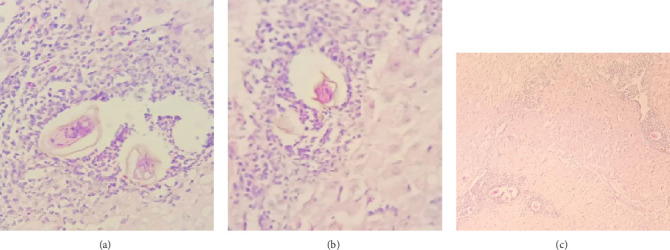
Two *Schistosoma haematobium* eggs surrounded primarily by lymphocytes (a). Prominent terminal spine characteristic of *Schistosoma haematobium*, which is more commonly associated with urogenital involvement compared to the lateral spines found on *Schistosoma mansoni*, a species more prevalent in the gastrointestinal tract (b). Low-power view (10 ×) shows a proliferation of luteinized cells arranged in a diffuse sheet pattern. These cells have abundant eosinophilic cytoplasm and round nuclei. Importantly, note the *Schistosoma* eggs embedded within the tumor (c).

Gross examination of the resected tissue revealed a mass with dark brown cut surfaces. Microscopically, the sections from the fallopian tube showed chorionic villi, extravillous trophoblasts, fibrin deposits, and inflammatory infiltrates, confirming the diagnosis of an ectopic pregnancy. The ovarian tissue showed normal ovarian structures with signs of luteinization. In particular, numerous eggs of *S. haematobium* were identified in the ovarian stroma and fallopian tube, surrounded by granulomatous inflammation consisting predominantly of lymphocytes and occasionally multinucleated giant cells (Figure 1).

Her immediate postoperative course was uneventful. The patient was discharged on the second postoperative day. She was informed of the histopathologic findings at the follow-up visit. Praziquantel was prescribed at a total dose of 60 mg/kg in three divided doses. She was informed about the possible effects of FGS on fertility and advised to seek prenatal care for future pregnancies.

## 3. Discussion

This case illustrates the important but often overlooked role of FGS in the reproductive morbidity of women living in schistosomiasis-endemic regions. The patient presented with a ruptured ectopic pregnancy and recurrent spontaneous abortions. Histopathologically, *S. haematobium* eggs were detected in the ovarian and fallopian tube tissue. With the absence of other apparent risk factors, it is assumed that her previous recurrent abortions and the ectopic pregnancy might be attributed to the *Schistosoma* infection.

FGS affects a significant number of women in endemic areas but remains underdiagnosed due to its insidious progression and nonspecific symptoms [17]. Studies have shown that up to 75% of women with urogenital schistosomiasis may have genital involvement [5, 18]. The true burden of FGS is likely underestimated due to limited access to diagnostic facilities and overlapping symptoms with other gynecological conditions.

Reproductive complications associated with FGS include infertility, subfertility, spontaneous abortions, and ectopic pregnancies [10, 13, 19]. The mechanisms by which FGS affects fertility are multifactorial and include anatomical damage due to fibrosis and scarring, immunological changes, and potential hormonal imbalances [20]. The deposition of *S. haematobium* eggs in the fallopian tubes leads to a granulomatous inflammatory reaction characterized by the infiltration of lymphocytes, plasma cells, and eosinophils and the formation of epithelioid cell granulomas [7]. Chronic inflammation leads to fibrosis and scarring and causes tubal obstruction, impaired ciliary function, and altered tubal motility [21]. These changes increase the risk of ectopic implantation of the embryo, as was the case in our patient.

In addition to mechanical obstruction, schistosome antigens can cause local immunosuppression and modulation of host immune responses, which may impair implantation and placental development [22]. This immunological aspect may contribute to recurrent pregnancy loss, although the exact mechanisms are not yet clear.

The diagnosis of FGS is challenging due to the absence of specific clinical features and the limitations of conventional diagnostic methods [23]. Urine microscopy for schistosome eggs lacks sensitivity for detecting genital involvement, as the shedding of eggs may be intermittent or absent in chronic infections [24]. Serological tests may indicate exposure but do not confirm active infection or organ involvement [25]. Direct observation of characteristic lesions through colposcopy or biopsy can aid diagnosis, although it is invasive and not readily available in resource-poor areas [26]. Histopathological examination of surgical specimens, as in our case, provides definitive evidence of schistosome eggs in genital tissues [17]. Recently, point-of-care (POC) lateral flow immunoassays (LFIAs) have been extensively developed and evaluated for the screening of schistosomiasis. Recent advancements have rapidly facilitated the creation of molecular-based POC diagnostics for schistosomiasis [27]. However, these POC tests are not available in our setting.

Praziquantel remains the cornerstone of schistosomiasis treatment due to its efficacy against adult worms of all *Schistosoma* species [28]. The World Health Organization recommends a single oral dose of 40 mg/kg for urogenital schistosomiasis, with retreatment considered for severe infections [2]. In our case, a total dose of 60 mg/kg, divided into three doses, was administered to increase efficacy. Early treatment is crucial to prevent disease progression and irreversible organ damage [29]. However, praziquantel cannot reverse existing fibrosis or scarring; therefore, early detection and intervention are crucial [30]. Treatment of reproductive complications may require surgical intervention, assisted reproductive technologies, or specialized obstetric care.

Addressing FGS requires a multifaceted public health approach, including mass drug administration; health education; and improving water, sanitation, and hygiene infrastructure [31]. Gender-specific interventions are needed to address the particular burden of FGS on women's health [32]. Training healthcare providers in the detection and treatment of FGS can improve diagnosis and treatment. Integrating schistosomiasis control into reproductive health services can improve accessibility and reduce stigma [33].

This case emphasizes the importance of considering FGS in women with unexplained reproductive problems in endemic areas. Clinicians should have a high suspicion of parasitic infection when evaluating cases of ectopic pregnancy, infertility, or recurrent pregnancy loss [34]. Histopathologic examination of resected tissues should be performed routinely, even if clinical suspicion of schistosomiasis is low. Knowledge of the characteristic morphological features of *S. haematobium* eggs, such as the terminal spine, can help pathologists to make a diagnosis [35].

## 4. Conclusion

FGS is a significant but often overlooked cause of reproductive morbidity in endemic regions. This case illustrates the presentation of FGS as a ruptured ectopic pregnancy in a woman with recurrent pregnancy loss. Clinicians should consider FGS in the differential diagnosis of reproductive health problems, particularly in women from endemic areas. Early diagnosis by histopathologic examination and prompt treatment with praziquantel are essential to prevent long-term sequelae. Public health efforts should focus on prevention, early detection, and education to mitigate the impact of FGS on women's health.

## Data Availability

All datasets generated and/or analyzed during the current study are included in this article.
